# Extraction and Characterization of Acidolysis Lignin from Turkey Oak (*Quercus cerris* L.) and Eucalypt (*Eucalyptus camaldulensis* Dehnh.) Wood from Population Stands in Italy

**DOI:** 10.3390/polym15173591

**Published:** 2023-08-29

**Authors:** Sara Bergamasco, Florian Zikeli, Vittorio Vinciguerra, Anatoly Petrovich Sobolev, Luca Scarnati, Giorgio Tofani, Giuseppe Scarascia Mugnozza, Manuela Romagnoli

**Affiliations:** 1 Department for Innovation in Biological, Agro-Food and Forest Systems (DIBAF), University of Tuscia, Via San Camillo de Lellis snc, 01100 Viterbo, Italy; zikeli@unitus.it (F.Z.); vincigue@unitus.it (V.V.); gscaras@unitus.it (G.S.M.); 2 “Annalaura Segre” Magnetic Resonance Laboratory, Institute for Biological Systems, CNR, Via Salaria, Km 29,300, 00015 Monterotondo, Italy; anatoly.sobolev@cnr.it; 3 Agenzia Regionale per lo Sviluppo e l’Innovazione dell’Agricultura nel Lazio—ARSIAL, Via Rodolfo Lanciani, 38, 00162 Roma, Italy; l.scarnati@arsial.it; 4 Department of Catalysis and Chemical Reaction Engineering, National Institute of Chemistry, Hajdrihova 19, 1000 Ljubljana, Slovenia; giorgio.tofani@ki.si

**Keywords:** acidolysis lignin, oak, eucalyptus, wood, HP-SEC, FTIR, Py-GCMS, 2D HSQC NMR spectroscopy, bioresources, short supply chain, bioeconomy

## Abstract

Acidolysis lignins from the species *Quercus cerris* L. and *Eucalyptus camaldulensis* Dehnh. were isolated and characterized using high pressure size exclusion chromatography (HP-SEC), Fourier-transform (FTIR) infrared spectroscopy, analytical pyrolysis–gas chromatography–mass spectrometry (Py-GCMS), and two-dimensional heteronuclear single quantum coherence (2D HSQC) NMR spectroscopy. The acidolysis lignins from the two different species varied in chemical composition and structural characteristics, with *Q. cerris* L. lignin having a higher S/G ratio and higher molar mass averages with a bimodal molar mass distribution. The different analytical techniques FTIR spectroscopy, Py-GCMS, and 2D NMR spectroscopy provided consistent results regarding the S/G ratio of the lignins from the two wood species. Based on the determined high S/G ratio of both oak and eucalypt lignin, the two wood sources could be promoted as substrates for efficient lignin isolation in modern forest biorefineries in order to develop innovative lignin-based value-added biorefinery products.

## 1. Introduction

The principle of a circular economy as a tool to establish a future green economy is of particular relevance when applied to the wood industry. Considering the forest-wood sector, the concept of cascading needs to be implemented as good practice, meaning that a processed wood product or wood processing residues are used at least once more as material or for energy generation [1]. Recently, Stafford et al. [2] highlighted the potential of forest biorefineries with their existing well developed infrastructure for the processing of huge amounts of biomass to develop new bioproduct value chains for product diversification, which could generate new revenues and improve their overall resilience. Furthermore, through the cascade use of wood in forest biorefineries, the global warming potential of these systems can be significantly reduced [3]. Forest biorefineries are characterized by a large portfolio of lignin- and cellulose-based products, such as biocomposites, resins, platform chemicals, building blocks for fine chemicals or bioactive compounds utilizing wood residues and wastes from the forest, timber, and pulp, and paper industry [2,4]. Additionally, there is a large potential in the feedstock supply considering the huge abundance of sawdust from sawing processes [4,5] and great volumes of different waste streams containing lignin, hemicelluloses, and extractives generated in the pulp and paper industry [2,6]. Among the most important components, lignin attracted high interest in recent times as it can be used for the production of several biomaterials [7,8,9]. The main reason for this interest is the fact that this biopolymer contains a broad range and high contents of chemical functionalities such as hydroxyl, carbonyl, and carboxyl groups [8,10,11,12,13]. The presence of these reactive functional groups allows for its utilization in biobased polymers development, such as a substitute for fossil-based compounds in polyurethane systems, polyesters, and epoxy resins [14,15,16].

One of the biggest challenges regarding the utilization of lignin is the fact that its molecular structure, properties, and reactivity are influenced by the original feedstock (softwood, hardwood and grass biomass) as well as the extraction process used for its isolation [17,18]. For this reason, the study of the chemical structure and presence of functional groups of lignins isolated from different plant sources is crucial to determine species-specific lignin properties and reactivity for possible eventual applications. 

The intention of this article is to valorize certain Italian local wood resources. The first one is Turkey oak (*Quercus cerris* L.), which is one of the most widespread deciduous oak species in Italy together with pubescent oak (*Q. pubescens* Willd) [19]. Turkey oak was used for railway sleepers and barrels in the past, but today it is mainly used as firewood. Wood chemistry studies regarding oak are not so frequent; some contributions are related to the interest for oak wood properties, for example in wine and spirit ageing [20]. The second short supply chain species investigated in this study is *Eucalyptus camaldulensis* Dehnh., which is present in many windbreaks in Italy. Eucalypt trees used for wind-breaking stripes in coastal agricultural areas protect crop cultivations from continuous stress due to constant winds and eventual water loss in the soil. *Eucalyptus* spp. Is frequently planted because of its high adaptability to different climatic conditions, fast growth, and high-level wood properties, i.e., in Brazil, 77% of all newly planted trees were from eucalypt species [5]. In Italy, there are more than 70,000 ha of eucalypt plantations and the most common species is *E. camaldulensis*, which was first described in 1832 from the botanical garden *Hortus Camaldulensis* near Naples, Italy. Besides the protection from soil erosion, eucalypt trees were massively introduced for the production of pulpwood for the Italian pulp and paper industry in the first half of the 20th century [21,22]. Because of the reduced importance of paper mills in Italy nowadays and limitations for high-value applications of eucalypt wood due to excessive shrinking and eventual splitting during drying causing low yields [23], eucalypt wood is often used for energy purposes as its caloric value is in the range of other common firewoods used in Italy like oak and beech [24]. A detailed knowledge of lignin structure of both species represents the first step for a rational exploitation of respective wood chips and wood processing residues for value-added future lignin applications. A wide range of analytical techniques has been used for the elucidation of lignin structural characteristics and the methods applied in the presented work are among the most important and most frequently used ones, namely FTIR and NMR spectroscopy, analytical Py-GCMS, as well as SEC analysis [17,19,25,26,27,28].

## 2. Materials and Methods

### 2.1. Materials

The studied oak wood was derived from a disk of an adult tree of *Q. cerris* from the Roccarespampani Estate close to Tuscania (Viterbo province, Italy). The eucalypt wood was derived from windbreak trees of *E. camaldulensis* close to Tarquinia (Viterbo province, Italy). The wood was obtained during tree management activities in 2022. For lignin extraction, the following solvents were used: acetone Re pure, 1,4-dioxane, hydrochloric acid 37%, dimethyl sulfoxide-d_6_ 99.5% (Isotopic), and potassium bromide (KBr) were purchased from Carlo Erba Reagents (Cornaredo, Italy). Polystyrene sulfonate standards were purchased from PSS Polymer Standard Services (Mainz, Germany), Millex^®^-GV syringe filters (0.22 µm) in PVDF were purchased from Millipore Corporation (Belford, MA, USA). Sodium hydroxide was purchased from Merck Life Science S.r.l. (Milan, Italy).

### 2.2. Lignin Isolation

Acidolysis lignins (AL) from oak and eucalypt were isolated using a mild acidolysis method, which was described in detail in earlier works of the group [27,28] and is based on the protocol of Gellerstedt et al. [29]. Prior to extraction, oak and eucalypt wood was physically pretreated, reducing it to a particle size of 0.25 mm using a cutting mill (MF 10 basic, IKA Werke, Staufen im Breisgau, Germany) and subsequently extracted with acetone-water (90:10, *v/v*) using a Soxhlet apparatus (24 g wood meal in 400 mL acetone-water). 

The extracted wood meal was collected, oven-dried at 40 °C (FD 115, Binder GmbH, Tuttlingen, Germany), and weighed. The extractives dissolved in acetone-water were brought to dryness and weighed as well to determine the extractives content of the used oak and eucalypt wood. The extracted biomass (13.5 g) was cooked under reflux using dioxane-water (400 mL) in a volumetric ratio of 82:18 in a 0.1 M HCl solution for two hours. At the end, the solution was filtered for removal of the acidolysis residue. The filtration residue was washed with a portion of 200 mL dioxane-water (82:18 vol%). In order to precipitate the dissolved lignin in the filtrate, dioxane was removed by rotary evaporation and replaced with water of equal volume fractions. Finally, the solution was filtered, and the collected lignin was further washed with acidified (pH 2) distilled water to remove sugar residues. Finally, the lignin was dried in a ventilated oven (J.P. Selecta, Barcelona, Spain) at 40 °C for 2 h and weighed to calculate the yield.

### 2.3. High Performance Size Exclusion Chromatography (HP-SEC)

The molecular weights of the extracted lignins were determined by HP-SEC. The analysis was performed using an Agilent 1100 HPLC system equipped with a temperated column compartment and a diode array detector using Agilent Chemstation (version B.03.02) for operation (Agilent Technologies Inc., Santa Clara, CA, USA). Molar mass analysis was performed using a PSS MCX column with 5 µm particle size and 1000 Å pore size (PSS Polymer Standard Services, Mainz, Germany) which was kept at 40 °C. The mobile phase was a 10 mM NaOH solution with addition of 20 mM NaNO_3_ as neutral salt. The flow rate was set to 0.5 mL/min and the UV signal at 280 nm was used for molar mass determination. For calibration, polystyrene sulfonate standards were used with the following molar masses at peak maximum: 65,400, 33,500, 15,800, 6430, 1670, 891, and 208 Da. Weight- and number-average molar masses were calculated using the GPC Add-on (version B.01.02) of Agilent Chemstation software.

### 2.4. Fourier Transform Infrared (FTIR) Spectroscopy

For FTIR analysis, potassium bromide (KBr) pellets containing 2 wt% of the respective lignin samples were prepared and measured against a background of pure KBr. Samples were prepared using a mini pellet press at 2 bar for 5 min (Specac Inc., Fort Washington, MD, USA). The FTIR spectra were acquired in absorbance mode with a resolution of 4 cm^−1^ and an average of 16 scans in a wavenumber range of 4000–800 cm^−1^ using a FTIR-4100 Fourier Transform Infrared spectrometer (Jasco Corp., Easton, MD, USA). The fingerprint region (1800–900 cm^−1^) was studied for each sample and the absorption bands were interpreted and assigned using reference literature reported in Table 3.

### 2.5. Pyrolysis–Gas Chromatography–Mass Spectrometry (Py–GCMS)

Samples of about 1.5 mg of the isolated AL from oak and eucalyptus wood were heated to 450 °C using a Pyrojector II (SCE Analytical Science, Trajan Scientific and Medical, Melbourne, Australia), connected to a GCMS-QP5050 instrument (Shimadzu Corporation, Kyoto, Japan). Helium was the carrier gas at a pressure of 100 kPa in the pyrolyzer and 70 kPa in the GC injector (280 °C, 1:20 split ratio). Furnace temperature was initially set at 45 °C for 4 min, then increased to 240 °C at a rate of 4 °C min^−1^, and finally to 280 °C at a rate of 39 °C min^−1^. The mass spectrometer was used in electron ionization (EI) mode at 70 eV and scans from *m*/*z* 35 to 500 were run in 0.7 s cycles. The pyrolysis products were identified by comparing their mass spectra with those of the NIST and Wiley libraries and those reported in literature as published in earlier works [26,27,28,30]. The peaks of interest were identified, the spectra were normalized to the most intense peak, and the respective peak areas were expressed as percentages.

### 2.6. Two-Dimensional (2D) HSQC NMR Spectroscopy

Two-dimensional ^1^H-^13^C heteronuclear single quantum coherence (2D HSQC) NMR spectra were recorded on a Bruker AVANCE 600 spectrometer operating at the proton frequency of 600.13 MHz with a z-gradient probe using 50 mg of the lignin samples in 700 µL DMSO-d_6_. The HSQC experiments were acquired using a time domain of 1024 data points in the F2 dimension (^1^H) and 512 data points in the F1 dimension (^13^C). A ^1^J_C-H_ coupling constant of 145 Hz and spectral widths of 12 ppm and 220 ppm in the ^1^H- and ^13^C-dimensions, respectively, were used applying 128 scans with a recycle delay of 3 s. For a semi-quantitative analysis, the integrals of the C-H cross-peaks were determined and compared using MestReNova v6.0.2 software (Mestrelab Research S.L., Santiago de Compostela, Spain). Signal assignment was conducted according to the literature [31,32,33,34,35,36,37]. The monomeric ratio of the AL fractions was estimated from the C_2_-H_2_ correlations from syringyl (S), guaiacyl (G), and *p*-hydroxyphenyl (H) lignin subunits as well as ferulic acid (FA) groups in the aromatic region of the HSQC spectra. The C_α_-H_α_ correlations were used for the determination of the relative abundances of the different lignin inter-unit linkages as well as the ratio of stilbene structures.

## 3. Results and Discussion

Table 1 shows the respective extractives and AL yields for Turkey oak and eucalypt wood in relation to the mass of the starting material. The determined extractives content was three times higher in eucalypt wood compared to oak, while the AL isolation yields were comparable in the two different wood species. Comparing these yields with the Klason lignin contents of the two species reported in literature, which can vary a lot depending on the source [38,39,40,41,42], it could be concluded that the acidolysis method, independent of the used wood species and its respective lignin content, has a maximum extraction efficiency of about 7% relative to the dry wood mass and about 30% of the respective Klason lignin content.

### 3.1. Molar Mass Distribution (MMD)

Analysis through HP-SEC offers a clear insight concerning the molecular weight distribution of extracted lignins, which enriches knowledge in terms of reactivity and chemical-physical properties of lignin since they are partly determined by their MMD. As a result of the extraction process, lignins are depolymerized into smaller fractions, and so HPSEC analysis can provide an estimate of the delignification and of pulping rate of a certain extraction process [43].

**Figure 1:**
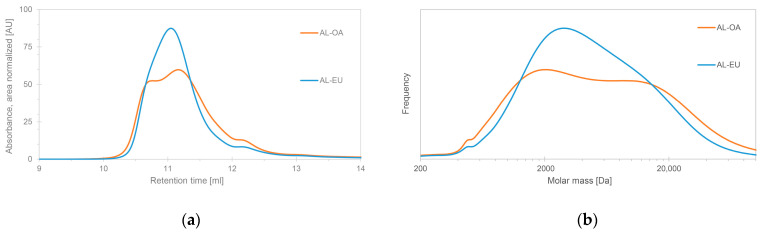
HP-SEC chromatograms (**a**) and molar mass distributions (**b**) of the isolated acidolysis lignins from oak (AL-OA) and eucalypt (AL-EU).

Figure 1 shows the HP-SEC chromatograms (left) of the two isolated acidolysis lignins AL-OA and AL-EU and their respective molar mass distributions (MMDs) (Figure 1b). While AL-OA shows a bimodal MMD, the elugram of AL-EU exhibits a broad single peak. The maximum of the elution curve of AL-EU is found in between the two maxima of AL-OA and the overall MMD appears slimmer, which reflects in the calculated dispersity (Đ, Table 2). Thus, the calculated weight-average molar mass (M_w_) of AL-OA is higher than the one of AL-EU while the number-average molar mass (M_n_) is lower.

Interestingly, the determined molar mass averages of AL-OA and AL-EU were a little higher than the results obtained for ALs isolated from beech and chestnut as well as iroko and mixed iroko and Norway spruce sawdust using the same protocol, which were re-analyzed under the same HP-SEC conditions and using the same software for molar mass determination [27,28]. Comparing those ALs from six different wood species, AL from oak wood had the highest value for M_w_ but also a high dispersity (Đ), which was in the range of AL from beech wood. High molar mass averages indicate little cleavage of lignin inter-unit linkages during isolation, which was an objective of the used mild acidolysis protocol in order to obtain rather native lignin samples. Further, the very intense cross-peak of the C_α_ atom assigned to *β*-O-4′ linkages (Figure 4) and the corresponding high relative abundance of *β*-O-4′ bonds (Table 5) support the conclusion that little lignin depolymerization occurred during acidolysis. Considering the high M_w_ as well as M_n_ of AL-EU, resulting in a rather low dispersity, AL from eucalypt wood could be considered as the one with the highest molar mass out of the selection of the six ALs from different wood species.

Molar mass value has an impact on lignin properties and its possible applications. For example, it was shown that lignin fractions with lower molecular weights, and as a consequence, exhibits stronger scavenging and antioxidant abilities with higher amounts of phenolic hydroxyl groups, making it a promising active ingredient in biomedical engineering applications [44].

### 3.2. FTIR Spectroscopy

**Figure 2:**
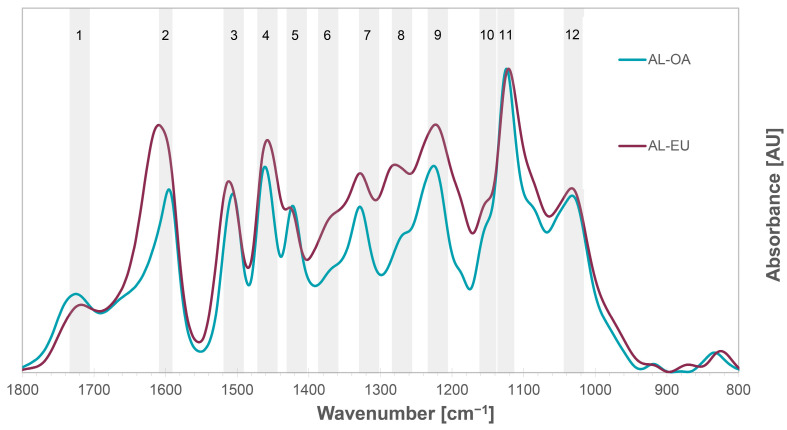
FTIR spectra of the isolated acidolysis lignins from oak (AL-OA) and eucalypt wood (AL-EU). The respective signal assignments of the IR band numbers are given in Table 3.

The FTIR spectra of AL-OA and AL-EU were of similar shape; specific differences of the IR bands intensities were observed. The chemical structures assigned to the respective characteristic IR absorbance bands in Figure 2 are listed in Table 3. The IR band 1 located at 1725–1719 cm^−1^ corresponds to the stretching vibration of carbonyl groups in unconjugated ketones, aldehydes, and esters in hemicelluloses. The low intensity of this IR band indicates a low contribution of hemicelluloses and thus a high purity of the prepared AL samples. Band 2 around 1611–1593 cm^−1^ was assigned to the aromatic skeletal vibration with contributions of carbonyl C=O stretching modes. There was a significant difference in IR band 2 between the AL samples of the two species with AL-EU showing a wider absorbance band with a maximum shifted to a higher wavenumber, most probably deriving from additional C=O stretching modes indicating a higher content of C_α_-oxidized structures in AL-EU. Therefore, a contribution to this signal could be the C_α_-oxidized structures which are present in both lignin samples, as observed by 2D NMR analysis (Table 5). However, the relative contents of C_α_-oxidized structures determined by 2D NMR spectroscopy were higher in AL-OA than in AL-EU, indicating further structures present in AL-EU, which cause the high absorbance in the region of IR band 2. Comparing the FTIR spectrum of AL-EU with the one of an AL isolated from iroko sawdust, which was apparently rich of contributions form extractives moieties [28], it could be speculated that there is also a considerable presence of extractives in the AL-EU isolated in this study. An additional explanation of the high IR absorbance at 1640–1610 cm^−1^ is given by Xiao et al. [34], who attribute this phenomenon to the presence of small molecular compounds like polyphenols, condensed tannins, or calcium oxalate crystals, which is the most common formed minerals in plants, inside the wood structure of eucalypt.

The absorption bands at 1512–1505 cm^−1^ (IR band 3) and at 1425–1422 cm^−1^ (IR band 4) correspond to aromatic ring breathing modes. The IR band between 1372–1367 cm^−1^ (IR band 6) represents aliphatic C–H stretching in methyl but not in methoxyl groups and phenolic O–H stretching vibration, as well as C-H bending deformation for cellulose and hemicelluloses. The low intensity of this band indicates a low content of free phenolic OH groups, which coincides with rather high molar masses determined by HP-SEC (Table 2) as well as low carbohydrate impurities. The band at 1328–1327 cm^−1^ is characteristic for aromatic ring breathing of syringyl moieties, while the IR band around 1282–1265 cm^−1^ corresponds to guaiacyl ring breathing, with additional contributions of C=O structures. The absorbance band at 1224–1223 cm^−1^ (IR band 9), instead, reflects both syringyl and guaiacyl ring breathing. A strong absorption band at 1125–1122 cm^−1^ stands for aromatic skeletal C-H in-plane deformations and C-O stretching modes and represents the most prominent IR band in the spectra of both AL samples. In addition, the IR band 12 at 1031 cm^−1^ corresponds to aromatic C–H stretching in-plane deformation and C=O stretching modes in lignin and cellulose. 

According to Faix et al. [48], these specific IR band intensities can be used for the classification of lignins into G and different types of GS lignins depending on the ratio of G and S units, respectively. The higher the ratio of S units, the more dominant the maximum at 1125 cm^−1^ (IR band 11). Considering that IR band 11 is the most prominent IR band in the spectra of both AL-OA and AL-EU lignins, it can be concluded that their respective S/G ratio is in favor of S units and both lignins could be characterized as lignins of type GS 4 according to Faix et al. [48]. The second requisite of this lignin type is that the intensity of IR band 4 is higher than the one of IR band 3, which is the case for both AL-OA and AL-EU. The bigger difference between A1462 (IR band 4) and A1505 (IR band 3) in the FTIR spectrum of AL-EU compared to the one of AL-OA indicates that the respective S/G ratio of AL-EU is higher than in AL-OA. The third characteristic is a higher intensity of IR band 7 than IR band 8 and that the intensity at 1268 cm^−1^ (IR band 8) is very low. This is the case for AL-OA but not for AL-EU, indicating that AL-OA is more dominated by S units than AL-EU and thus the S/G ratio of AL-OA should be higher than the one of AL-EU. Similar observations were reported also by Xiao et al. [34], who identified their isolated eucalypt lignins as typical GS lignins with rather high S units content based on the acquired FTIR spectra. 

### 3.3. Analytical Py-GCMS

**Figure 3:**
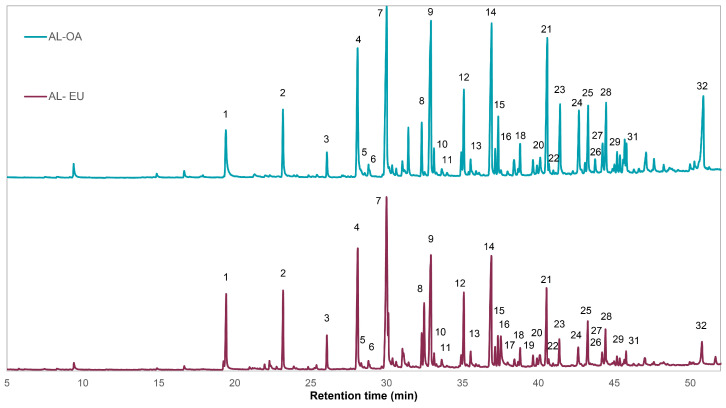
Pyrograms of the isolated acidolysis lignins from oak (AL-OA) and eucalypt wood (AL-EU). Peak numbers refer to the pyrolysis products listed in Table 4.

Analytical Py-GCMS is a method that allows to study the complex structure of lignin after thermal degradation of the polymer by destruction of certain chemical bonds, leading to the formation of a mixture of volatile aromatic compounds that are separated by GC and finally analyzed by MS. The pyrograms of AL-OA and AL-EU showed similar profiles, and pyrolysis products were identified (Figure 3, Table 4). Among the phenolic compounds, mainly compounds derived from guaiacol (G) and syringol (S) units were found. Usually the S/G ratio of hardwood lignins is expected to be over 1, due to the higher abundance of syringyl moieties in hardwoods in general. However, different studies observed a great variability in hardwoods S/G ratios with values < 1, such as in *Eucalyptus tereticornis* (0.7), *Acer macrophyllum* (0.51), or in *A. negundo* (0.4), as pointed out by Vinciguerra et al. [26]. Variations of the S/G ratio are also possible within the same species like for chestnut lignin where Vinciguerra et al. [26] determined values between 1.29 and 3.49, while in a later work an S/G ratio for chestnut AL of 0.56 was observed [27]. In the case of AL-EU, the highest abundance was observed for 4-vinylguaiacol, syringol, 4-methylsyringol, and 4-vinylsyringol and the respective S/G ratio for AL-EU was 2.11, corresponding with literature values for *Eucalyptus globulus* as well as *E. camaldulensis* lignins [42,53]. Regarding AL-OA, slightly different peak areas of the pyrolysis products were identified and the respective S/G ratio was 2.73, which is in good concordance with the S/G ratio determined for dioxane lignin by [20] using 2D HSQC NMR spectroscopy. In the case of AL-OA, the main pyrolysis products were 4-vinylguaiacol, syringol, 4-methylsyringol, 4-vinylsyringol, 4-propenylsyringol, and sinapaldehyde. There was one pyrolysis product, 4-oxy-allylsyringol, which was detected exclusively in the pyrogram of AL-OA. According to literature reports, hardwoods with a higher lignin S/G ratio can be processed with more efficiency, which brings a higher lignin isolation yield due to stronger delignification during pulping [54]. Therefore, both *E. camaldulensis* and *Q. cerris* can be considered as preferred starting materials for an efficient lignin isolation, which is the base for the development of a cost-effective and sustainable lignin valorization route.

### 3.4. 2D NMR Spectroscopy

**Figure 4:**
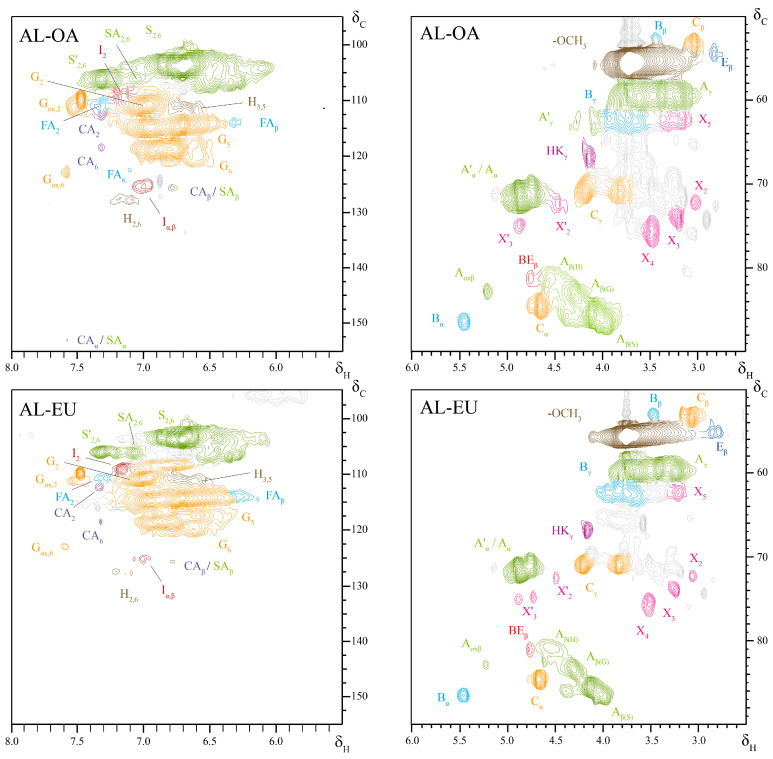
2D HSQC NMR spectra of the acidolysis lignins isolated from oak and eucalypt wood (AL-OA, AL-EU). Left: aromatic regions. Right: oxygenated side-chain regions. For the corresponding chemical structures and respective NMR shifts, see Figure 5 and Table 6.

**Figure 5:**
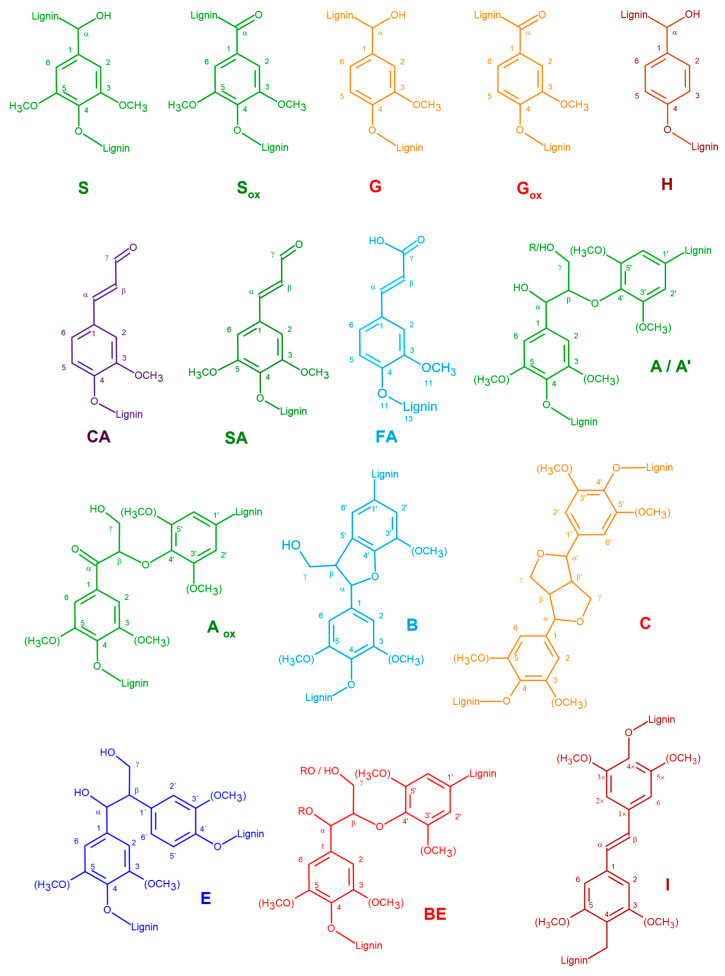
Lignin substructures identified in the respective 2D HSQC NMR spectra of oak and eucalypt acidolysis lignin. S … syringyl units, S_ox_ … *α*-oxidized S units, G … guaiacyl units, G_ox_ … *α*-oxidized G units, H … *p*-hydroxyphenyl units, CA … coniferaldehyde, SA … sinapaldehyde, FA … ferulic acid, A … *β-*O-4′ linkages, A’ … acylated *β-*O-4′ linkages, A_ox_ … *α*-oxidized *β-*O-4′ linkages, B … *β*-5′ phenylcoumaran bonds, C … *β*-*β′* resinol bonds, I … stilbene structures, BE … benzyl ether structures, E … open *β*-1′ structures.

Both oak and eucalypt ALs showed a monomeric composition almost exclusively of S and G units with minimal contributions of H units and FA groups (Table 5 and Figure 4). The respective structures are illustrated in Figure 5. Interestingly, the corresponding C_2_, C_6_, and C_β_ cross-peaks (FA_2_, FA_6_, and FA_β_) for FA were found, while the C_α_ cross-peak was not detected. Moreover, the NMR spectra show the presence of polysaccharides contamination (X_n_ and X’_n_). In AL-OA, S units were more abundant than in AL-EU, resulting in a higher S/G ratio. Both lignins were mainly connected through *β*-O-4′ bonds, which accounted for around 75–82% when including also α-oxidized *β*-O-4′ substructures. AL-OA showed about 10% more *β*-O-4′ interunit linkages and fewer phenylcoumaran structures, which could come as a consequence of a higher S units ratio. Resinol (*β*-*β*′) structures were more abundant than phenylcoumaran-type interunit linkages (*β*-5′) in both lignins, most probably caused by the strong presence of S units which do not allow for phenylcoumaran-type interunit linkages. Further, benzylether (BE), open *β*-1′, stilbene (I) structures, and cinnamaldehyde (CA) end groups were identified in the 2D NMR spectra of the two different lignins. The determined S/G ratio of the isolated oak AL was higher than the values reported by Lourenco et al. [31] for the lignin of cork oak xylem isolated after ball-milling using dioxane-water (2.31 vs. 1.2–1.6), but in good accordance with the one reported by Vivas et al. [20], as already mentioned above. On the other hand, the S/G ratio of eucalypt AL was close to the one from *E. camaldulensis* milled wood lignin (MWL) reported by Wang et al. [40] as well as in the range of the S/G ratios reported for *E. camaldulensis* by Kawamura et al. [42]. Other studies found considerably higher S/G between 2.7 and 3.45 for an enzymatically isolated lignin (SREL) from *Eucalyptus urophylla* × *E. grandis* [34]. However, it was also shown that the S/G ratio of an SREL preparation was considerably higher than the one of an MWL from the same starting material [40]. Since the isolation protocol applied in this study involves the same solvent as used for MWL isolation, it can be expected that the isolated AL fraction also contains the MWL of the processed eucalypt wood and therefore the S/G ratio was found in the range of a eucalypt MWL, as mentioned above. The AL samples of the different wood species showed differences in their acylation degree of lignin sidechains. While for AL-EU no cross-peaks for C_γ_ acylated lignin sidechains were detected (A’_γ_), the respective signals were identified in the spectrum of AL-OA.

## 4. Conclusions

The structural and chemical differences between oak and eucalypt lignins from the same extraction process were highlighted. As the lignins from the two wood species showed rather different structures, the detected differences could be considered as intrinsic to their respective nature. In particular, different analysis techniques, such as FTIR spectroscopy, Py-GCMS, and NMR spectroscopy, delivered consistent results, confirming that AL-OA has a higher S/G ratio than AL-EU. As the isolated lignins of both species exhibit high S/G ratios, they qualify as preferred lignin extraction sources due to higher extraction efficiency based on their high abundance of S units. Two-dimensional NMR spectroscopy indicated a rather homogenous and linear lignin structure based on a high ratio of *β-*O-4′ substructures, and FTIR spectroscopy indicated AL fractions with low carbohydrates impurities although 2D NMR revealed xylan contributions in the lignin macromolecule. Molar mass analysis showed rather small molecular weights of the isolated lignins, indicating their suitability for application as antioxidant or radical scavengers considering that these activities increase with lower molar mass. Knowledge of the chemical properties and the complex molecular structure of the species-specific lignins provides important information in order to valorize the wood resources studied in the presented work for the development of sustainable and competitive products in different value-added application fields. Further studies regarding purity and a quantification of functional groups contents (phenolic, aliphatic, and carboxylic hydroxyl groups) using ^31^P-NMR spectroscopy will provide useful data in order to define clearer future application fields of the isolated lignins.

## Figures and Tables

**Table 1:** Extractives content and acidolysis lignin (AL) isolation yield as well as Klason lignin contents for oak (*Q. cerris*) and eucalypt (*E. camaldulensis*) wood reported in reference literature [38,39,40,41,42].

Wood Species	Extractives (%)	AL (%)	Klason Lignin (%)
*Q. cerris*	2.0	7.3	23.2–25.5
*E. camaldulensis*	6.1	7.2	21.4–27.7

**Table 2:** Weight-average (M_w_) and number-average (M_n_) molar mass, and dispersity (Đ) of the isolated acidolysis lignins from oak and eucalypt wood (AL-OA and AL-EU). Acidolysis lignins from beech wood (AL-B), chestnut sawdust (AL-Ch), iroko (AL-IR), and mixed iroko and Norway spruce sawdust (AL-IRNS) from earlier works are also reported.

LIGNIN SAMPLE	M_w_ (Da)	M_n_ (Da)	Đ
AL-OA	5170	1227	4.21
AL-EU	4841	1472	3.29
AL-B	4510	1032	4.37
AL-Ch	3583	1099	3.26
AL-IR	3270	1084	3.02
AL-IRNS	3895	1126	3.46

**Table 3:** IR bands and assigned chemical structures found in the lignin samples.

IR Band	Wavenumber (cm^−1^)		Assignment	Reference
	Oak	Eucalypt		
1	1725	1719	C=O stretch in unconjugated ketones, in carbonyl and ester group	[45,46]
2	1593	1611	Aromatic skeletal vibrations plus C=O stretching, C=C aromatic ring vibration (S > G), G condensed > G etherified	[45,47,48]
3	1505	1512	C=C aromatic ring vibration (G> S)	[49]
4	1461	1459	C-H deformation in methyl and methylene groups	[48]
5	1422	1425	C=C aromatic ring vibration and C-H in plane deformation	[45]
6	1367	1372	Aliphatic C-H stretching in CH3 (not in OCH3) and phenolic O-H stretching vibration, C-H bending vibration in cellulose and hemicelluloses	[47,50]
7	1328	1327	Syringyl ring breathing with C-O stretching	[49]
8	1265	1282	Guaiacyl ring breathing, C=O stretching in lignin and C-O linkage in guaiacyl aromatic methoxyl groups	[49]
9	1224	1223	C-C plus C-O stretching and C=O stretching; G condensed > G etherified, syringyl and guaiacyl ring breathing	[51,52]
10	1148	1150	C-H stretching in aromatic ring (guaiacyl)	[49]
11	1125	1122	Aromatic skeletal and C-O stretching (typical for S units)	[48,50]
12	1031	1031	Aromatic C-H in-plane deformation in guaiacyl and C–O deformation in primary alcohol	[48,50]

**Table 4:** Relative abundance of pyrolysis products of the isolated acidolysis lignins from oak (AL-OA) and eucalypt (AL-EU).

Peak Number	Pyrolysis Product	Origin	RT (min)	AL–OA	AL–EU
				Area (%)	Area (%)
1	Guaiacol	G	19.42	2.39	3.50
2	4-Methylguaiacol	G	23.17	2.43	3.30
3	4-Ethylguaiacol	G	26.09	0.80	1.34
4	4-Vinylguaiacol	G	28.09	6.24	7.08
5	Eugenol	G	28.85	0.34	0.28
6	4-Propylguaiacol	G	28.86	0.15	0.15
7	Syringol	S	29.99	9.47	13.06
8	Isoeugenol (trans)	G	32.30	1.61	1.35
9	4-Methylsyringol	S	32.91	8.00	7.84
10	Vanillin	G	33.11	0.77	0.57
11	Homovanillin	G	34.90	0.82	0.59
12	4-Ethylsyringol	S	35.08	2.85	3.46
13	Acetoguaiacol	G	35.52	0.70	0.91
14	4-Vinylsyringol	S	36.91	7.67	7.67
15	Guaiayl acetone	G	37.15	0.70	0.79
16	4-Allylsyringol	S	37.34	1.69	1.21
17	Propioguaiacone	G	37.90	0.17	0.44
18	Coniferyl alcohol (structure isomer)	G	38.39	0.69	0.41
19	4-Propenylsyringol (cis)	S	38.78	0.98	0.77
20	Levoglucosan	PS	40.11	0.97	1.00
21	4-Propenylsyringol (trans)	S	40.55	6.29	3.85
22	Dihydroconiferyl alcohol	G	40.67	0.44	0.40
23	Syringaldehyde	S	41.39	2.86	1.32
24	Homosyringaldehyde	S	42.66	2.50	1.05
25	Acetosyringone	S	43.26	2.43	2.20
26	Coniferyl alcohol (cis)	G	43.74	0.50	0.04
27	Coniferaldehyde	G	44.21	1.15	0.70
28	Syringyl acetone	S	44.43	2.51	1.66
29	Propiosyringone	S	54.17	0.60	0.37
30	4-Oxy-allylsyringol	S	45.57	0.41	---
31	Sinapyl alcohol (structure isomer)	S	45.66	0.98	0.25
32	Sinapaldehyde	S	50.81	5.36	1.43
	**S/G Ratio**			**2.73**	**2.11**

**Table 5:** Monomeric ratios, S/G ratios, and relative abundance of lignin inter-unit linkages of the Acidolysis lignins from oak (AL-OA) and eucalypt (AL-EU).

Lignin Sample	Monomers (%)									Interunit Linkages (%)						
	S Units	Sox Units	G Units	Gox Units	H Units	SA	CA	FA	S/G Ratio	*β*-O-4′	*α*-oxid. *β*-O-4′	*β*-5′	*β*-*β*′	BE	Open *β*-1	Stilbenes
AL-OA	62	5	24	5	1	1	1	1	2.31	79	3	4	7	3	2	2
AL-EU	57	3	33	3	1	2	0	1	1.69	73	2	6	8	5	4	2

**Table 6:** Signal assignments of the ^13^C−^1^H correlation peaks in the 2D HSQC NMR spectra of the isolated wheat straw lignin fractions, according to [31,32,33,34,35,36,37]. The labels correspond to the respective lignin substructures shown in Figure 4.

δC/δH (ppm)	Assignment (Label)
52.7/3.43	C*_β_*-H*_β_* in phenylcoumaran *β*-5′ substructures (**B**_β_)
53.3/3.05	C*_β_*-H*_β_* in resinol substructures *β*-*β′* (**C_β_**)
54.2/2.82	C*_β_*-H*_β_* in open *β*-1′ substructures (**E_β_**)
59.6/3.05–3.93	C*_γ_*-H*_γ_* in *γ*-hydroxylated *β*-O-4′ substructures (**A_γ_**)
62.1/3.17	C_5_-H_5_ in β-D-xylopyranoside (**X_5_**)
62.5/3.76	C*_γ_*-H*_γ_* in phenylcoumaran *β*-5′ substructures (**B_γ_**)
62.5/4.29	C*_γ_*-H*_γ_* in *γ*-acylated *β*-O-4′ substructures (**A_γ_′**)
66.9/4.14	C*_γ_*-H*_γ_* in Hibbert’s ketone (**HK_γ_**)
70.8/3.80 + 4.15	C*_γ_*-H*_γ_* in resinol substructures *β*-*β′* (**C_γ_**)
71.5/4.84	C*_α_*-H*_α_* in *β*-O-4′ substructures (**A_α_**) linked to a G unit
74.1/3.02	C_2_-H_2_ in β-D-xylopyranoside (**X_2_**)
72.6/4.49	C_2_-H_2_ in 2-O-acetyl-β-D-xylopyranoside (**X′_2_**)
74.7/3.23	C_3_-H_3_ in β-D-xylopyranoside (**X_3_**)
75.5/4.86	C_3_-H_3_ in 3-O-acetyl-β-D-xylopyranoside (**X′_3_**)
76.0/3.48	C_4_-H_4_ in β-D-xylopyranoside (**X_4_**)
81.5/4.73	C*_α_*-H*_α_* in benzyl ether substructures (**BE_α_**)
81.6/4.53	C*_β_*-H*_β_* in *β*-O-4′ substructures (**A_β(H)_**) linked to a H unit
83.1/5.20	C*_β_*-H*_β_* in α-oxidized (C_α_=O) *β*-O-4′ substructures (**A_oxβ_**)
83.4/4.28	C*_β_*-H*_β_* in *β*-O-4′ substructures (**A_β(G)_**) linked to a G unit
85.0/4.67	C*_α_*-H*_α_* in resinol *β-β′* substructures (**C_α_**)
86.2/4.01	C*_β_*-H*_β_* in *β*-O-4′ substructures (**A_β(S)_**) linked to a S unit
87.1/5.50	C*_α_*-H*_α_* in phenylcoumaran *β*-5′ substructures (**B_α_**)
103.4/6.64	C_2,6_-H_2,6_ in syringyl units (**S_2,6_**)
106.2/7.06	C_2,6_-H_2,6_ in sinapaldehyde units (**SA_2,6_**)
106.2/7.30	C_2,6_-H_2,6_ in α-oxidized (C_α_=O) syringyl units (**S′_2,6_**)
110.6/7.32	C_2_-H_2_ in ferulic acid (**FA_2_**)
110.6/7.51	C_2_-H_2_ in oxidized guaiacyl units (**G′_2_**)
110.8/6.97	C_2_-H_2_ in guaiacyl units (**G_2_**)
111.8/6.66	C_3,5_-H_3,5_ in *p*-hydroxyphenyl units (**H_3,5_**)
112.9/7.33	C_2_-H_2_ in coniferaldehyde (**CA_2_**)
115.2/6.80	C_5_-H_5_ in guaiacyl units (**G_5_**)
119.3/6.88	C_6_-H_6_ in guaiacyl units (**G_6_**)
118.4/7.32	C_6_-H_6_ in coniferaldehyde (**CA_6_**)
123.0/7.11	C_6_-H_6_ in ferulic acid (**FA_6_**)
122.8/7.62	C_6_-H_6_ in oxidized guaiacyl units (**G′_6_**)
125.6/7.01	C*_α_*_,*β*_-H*_α_*_,*β*_ in stilbene units (**I*_α_*_,*β*_**)
126.0/6.77	C*_β_*-H*_β_* in cinnamyl aldehyde end groups (**CA***_β_*)
128.1/7.14	C_2,6_-H_2,6_ in *p*-hydroxyphenyl units (**H_2,6_**)
153.6/7.60	C*_α_*-H*_α_* in cinnamyl aldehyde end groups (**CA**_α_, **SA_α_**)

## Data Availability

The data presented in this study are available on request from the corresponding authors.
